# Toward the Next Generation of Passive Micromixers: A Novel 3-D Design Approach

**DOI:** 10.3390/mi12040372

**Published:** 2021-03-30

**Authors:** Mahmut Burak Okuducu, Mustafa M. Aral

**Affiliations:** 1 School of Civil and Environmental Engineering, Georgia Institute of Technology, Atlanta, GA 30332, USA; 2 Design and Simulation Technologies Inc., Istanbul 34860, Turkey; mmaral@live.com

**Keywords:** micromixer, diffusive mixing, passive mixing, fluid overlapping, sequential injection, segmentation, concentric flow, CFD

## Abstract

Passive micromixers are miniaturized instruments that are used to mix fluids in microfluidic systems. In microchannels, combination of laminar flows and small diffusion constants of mixing liquids produce a difficult mixing environment. In particular, in very low Reynolds number flows, e.g., Re < 10, diffusive mixing cannot be promoted unless a large interfacial area is formed between the fluids to be mixed. Therefore, the mixing distance increases substantially due to a slow diffusion process that governs fluid mixing. In this article, a novel 3-D passive micromixer design is developed to improve fluid mixing over a short distance. Computational Fluid Dynamics (CFD) simulations are used to investigate the performance of the micromixer numerically. The circular-shaped fluid overlapping (CSFO) micromixer design proposed is examined in several fluid flow, diffusivity, and injection conditions. The outcomes show that the CSFO geometry develops a large interfacial area between the fluid bodies. Thus, fluid mixing is accelerated in vertical and/or horizontal directions depending on the injection type applied. For the smallest molecular diffusion constant tested, the CSFO micromixer design provides more than 90% mixing efficiency in a distance between 260 and 470 µm. The maximum pressure drop in the micromixer is found to be less than 1.4 kPa in the highest flow conditioned examined.

## 1. Introduction

Over the past few decades, improvements in microfabrication technology [1] and successful implementation of complex processes at microscales [2] have led the development of microfluidic systems. Micro total analysis systems (µTAS) or lab-on-a-chip (LOC) devices [3,4] are commonly referred to describe a centimeter-size compact unit in which physical, chemical, and biological processes take place in a microchannel network. Micromixers are typically one of the major operational sub-units of microfluidic schemes [5] and are employed to mix fluids using active or passive [6,7,8,9] mixing principles. In active mixing, extra modules are required to generate external disturbance forces on the flow domain (e.g., electrical, thermal, magnetic, acoustic, and pressure [10,11,12]) which develops a complexity in terms of fabrication and integration of these components with other microchip elements [11,13,14]. On the contrary, despite offering a relatively poor mixing performance, passive micromixers are simple devices with notable structural and operational advantages. In passive mixing approach, fluid mixing is carried out by exploiting the fluid flow energy within the micromixer and two well-known mixing phenomena: advection and molecular diffusion [4]. As such, additional module and power source requirements are eliminated, and mixing performance is mainly governed by micromixer geometry. Therefore, passive micromixers are typically easy to fabricate, provide simple and stable operating conditions, and offer better integrability [1,6,15], which make these devices prevalent to mix fluids at microscales.

In passive micromixers, fluid mixing arises as a challenging work due to advection-dominant transport developed in microchannels. Strictly laminar fluid flow that is usually Reynolds (Re) number << 100 [16,17] and very low molecular diffusion coefficients (e.g., typically in the range of 10^−9^–10^−11^ m^2^/s [12]) fundamentally create tough mixing conditions. These difficulties are generally dealt with generating a so-called chaotic fluid motion in special micromixer designs that is typically achieved when Re > 10–20. Thus, contact surface area between fluids is enhanced, which in turn expedites diffusive mixing. Typical design and fluid mixing examples may be seen in References [18,19,20,21]. At very low Re numbers (e.g., Re < 5–10); however, the effective use of advection is inhibited drastically due to unidirectional fluid flow developed in microchannels. In this case, mixing length increases significantly since mixing process mainly depends on molecular diffusion which is a slow process. Therefore, a long mixing channel is required to obtain an adequate mixing efficiency (e.g., 80% [22]). Increase in mixing length induces three major problems in microfluidic systems as follows: the integration of the micromixer into a microfluidic scheme, high energy requirement, and a long mixing time.

To date, several passive micromixer geometries have been devised to improve the degree of fluid mixing over a short distance. Although majority of these efforts enhance fluid mixing by developing secondary flows when Re > 10–20, usually a considerable mixing length increase is observed at very low flow conditions. For instance, Gidde et al. [8] studied a planar passive micromixer with circular and square mixing chambers. The authors performed a wide range of Re number scenarios between 0.1 and 75 and showed that fluid mixing is promoted based on the development of chaotic advection. In both designs, mixing efficiency was improved noticeably when the flowrate increased, and the highest values were achieved at Re = 75. The distance that is required to yield 80% mixing efficiency is reported as approximately 3500, 9000, 11,000, and 5000 µm for Re = 0.1, 1, 5, and 75, respectively. A similar behavior is also observed in spiral, omega, and interlocking semi-circular shape passive micromixer designs in [23]. While all the three designs perform well beyond Re = 1, mixing efficiencies follow a decreasing trend with rising flowrates between Re = 0.01 and 1. In the article [23], this is related to the short residence time of fluids and inadequate Dean vortices developed in curved microchannels. Although the overall channel length is around 22,000 µm in the spiral micromixer design, mixing efficiencies vary between 65–70% for the Re number range of 0.01–1. Bhagat et al. [24] examined the effects of several obstruction geometries in the mixing channel of a Y-shape micromixer. While obstructed micromixer provides almost a complete fluid mixing at Re = 0.01 (measured at ~5000 µm axial distance), roughly 20% and 40% efficiency drop is observed at Re = 0.1 and 1, respectively. Nonetheless, further increase of flowrate after Re = 1, which is defined as an inflection point in the study, increases the mixing performance by means of chaotic advection formed in the mixing channel. In our previous work [18], a similar inflection point was seen at Re = 5 after which the effective use of advection was accelerated in convex semi-circular ridge (CSCR) micromixer design. Although the CSCR micromixer geometry developed a complex flow profile even at very low flow conditions (e.g., Re ≤ 5), and hence enlarged the contact surface between fluids, short residence time of fluids reduced the diffusive activity across the interfacial area formed. Thus, a diminishing mixing efficiency profile was observed between Re = 0.1 and 5.

In several other studies, researchers focused on improving fluid mixing specifically at very low Re numbers. Fang et al. [25] studied a T-shape passive micromixer with several baffle-embedded mixing units along a mixing channel. Numerical and experimental results showed that the design proposed increased mixing performance by inducing chaotic advection in mixing units at Re = 0.29. Based on the simulation and experimental outcomes, it was reported that 28-period mixing unit is required (i.e., ~1.7 cm axial distance) to mix fluids completely. Ortega-Casanova and Lai [26] surveyed the effects of multiple inlets on mixing. The authors applied alternative inlet combinations on a passive micromixer design which has a single mixing unit similar to the one used in the study above [25]. Numerical outcomes show that increasing the number of inlets is more effective for the most challenging flow and transport conditions simulated in the study. When the effects of seven– and two–inlet configurations are compared, seven–inlet configuration increases mixing efficiency approximately 5.5 and 3.5 times for Re = 0.29 and 0.1 flow conditions, respectively. Mixing efficiencies quantified at the exit of the seven–inlet design (i.e., ~5000 µm axial distance) were reported as around 90% and 80% in Re = 0.1 and 0.29 flow cases, respectively. Lin et al. [21] proposed a 3-D circular passive micromixer design to generate vortex effects under low flow conditions. Using eight equally spaced inlets, a rotational fluid motion is created in the circular microchamber beyond a critical Re number that is 2.32. While the mixing efficiency of the micromixer is around 50% and 74% at Re = 0.5 and 2.32, respectively, more than 90% mixing values are obtained when Re ≥ 4 (measured at a ~1000 µm distance). Although vortex formation accelerated diffusive interaction between fluid bodies, high mixing efficiencies could also be reached as a result of using a relatively high molecular diffusion constant in the test cases, which is on the order of 10^−7^ m^2^/s. Goullet et al. [27] surveyed fluid mixing in a classical T-shape micromixer under sinusoidal fluid injection conditions at Re = 0.3. Results indicate that sinusoidal injection of fluids forms sequential fluid segments in the mixing channel, which in turn enhance contact surface and diffusive mixing. The degree of mixing was reported as 38.4, 70.3, and 86.5% (measured at 500 µm axial distance) for the injection frequencies of 1.25, 5, and 20 Hz, respectively.

As discussed above, mixing performance can be boosted in virtue of complex flow patterns which are mostly generated at relatively high Re numbers. When, however, very low Re conditions are examined, a substantial decrease in mixing performance is seen because of yielding a small contact surface between fluids. This is mainly caused by ineffective flow manipulations in microchannels. Although some injection and design strategies help to improve mixing at low Re numbers, overall micromixer length can still rise to the centimeter level which is not desired as noted earlier. In the present paper, we propose a novel fluid overlapping passive micromixer design to surpass the small interfacial area restrictions at very low fluid flow conditions. Unlike the conventional micromixer configurations, where the effective use of advection process is prioritized to enlarge contact surface, the novel design proposed enables forming a predefined interfacial area between fluid bodies in a compact geometry. Therefore, a rapid inter-diffusion between fluids is ensured, and mixing distance is decreased significantly.

## 2. Governing Equations and Mathematical Methods

Computational Fluid Dynamics (CFD) simulations are performed to numerically describe the fluid flow and scalar transport in the micromixer. In all scenarios tested, it is assumed that the system is isothermal and gravitational effects are negligible. Fluids to be mixed are of constant density and viscosity, miscible, and non-reactive. Based on the assumptions above, flow field is simulated using incompressible Navier-Stokes (NS) and continuity equations as presented in Equations (1) and (2), respectively. Advection–diffusion (AD) equation is employed to resolve the scalar transport domain in the micromixer as given in Equation (3).

(1) $$\rho \left[\frac{\partial u}{\partial t}+u\cdot \nabla u\right]=-\nabla p+\mu \nabla^{2}u$$

(2) ∇⋅u=0

(3) $$\frac{\partial c}{\partial t}+u\cdot \nabla c=D\nabla^{2}c$$

where *µ* is the viscosity (Pa∙s), *p* is pressure (Pa), *ρ* is the density (kg/m^3^), **u** = (u_x_, u_y_, u_z_) is the velocity vector (m/s), *c* is scalar concentration, and *D* is molecular diffusion coefficient (m^2^/s).

Equations (1)–(3) are solved using OpenFOAM [28] (v5.0, OpenFOAM Foundation, OpenCFD Ltd., Bracknell, UK, 2015) CFD package, in which governing equations are discretized based on finite volume method (FVM). While *simpleFOAM* and *scalarTransportFOAM* solvers are employed to simulate steady-state incompressible fluid flow and passive scalar transport, respectively, a modified form *icoFOAM* solver is exploited in time-dependent solutions of coupled flow and scalar transport equations. Solvers are arranged to treat the advection and diffusion terms in the governing equations with second-order accurate upwind [29] and central difference numerical schemes, respectively. In the *simpleFOAM* solver, SIMPLEC (semi-implicit method for pressure linked equations-consistent) [30] algorithm is used to solve pressure-velocity coupling. To ensure stability in steady-state simulations, under-relaxation technique [31] is used with a factor of 0.9. Steady-state solutions are accepted to be converged when residuals reach 1 × 10^−6^ threshold as typically practiced in several numerical micromixer studies [18,32,33]. In time-dependent simulations, temporal terms are discretized using Crank-Nicolson scheme with a blending coefficient of 0.9.

Dimensionless Reynolds (Re) and Peclet (Pe) numbers are used to determine the characteristics of fluid flow (e.g., laminar or turbulent) and scalar transport (e.g., advection– or diffusion–dominant) in the micromixer, respectively. Re and Pe numbers are computed in the exit channel of the micromixer as defined in Reference [34]. In the present study, flowrate-averaged mixing approach [33] is employed to quantify the degree of mixing on a given plane as formulized in Equation (4). Unmixed and fully mixed conditions are specified in a mixing index (MI) scale between 0 and 1, respectively. It should be noted that computing a mixing efficiency only relying on the scalar concentration distribution on a certain cross-section may provide imprecise mixing outcomes in cases where the distribution of scalar concentration is non-uniform in a flow profile. More information on this point can be found in References [18,33].

(4) $$MI=1-\sqrt{\frac{\sigma^{2}}{\sigma_{\max}^{2}}},\;\sigma^{2}=\frac{\int_{A}{\left(c-\bar{c}\right)}^{2}\cdot udA}{\int_{A}udA},\;\bar{c}=\frac{\int_{A}c\cdot udA}{\int_{A}udA}$$

where *A* is area, *u* is velocity, *σ* and *σ*^2^ are variance and the maximum variance, respectively, *c* is concentration, and $\bar{c}$ is the average concentration. In grid independence tests, Equation (5) is used to measure numerical discrepancy between a mesh density and the finest mesh for a given parameter.

(5) $$\Delta D_{M\text{-}F}=\left|\frac{P_{M}-P_{F}}{P_{F}}\right|\times 100$$

where *P_M_* and *P_F_* denote the parameter values, obtained from the numerical solutions of a certain mesh density and the finest mesh, respectively. Δ*D_M-F_* shows the difference, as a percentage, between a mesh level and the finest mesh with respect to the parameter employed. In this research, pressure drop (Δ*p*) and mixing efficiency (MI) parameters are used to assess numerical errors in fluid flow and scalar transport simulations, respectively.

## 3. Micromixer Design and Simulation Setup

**Figure 1:**
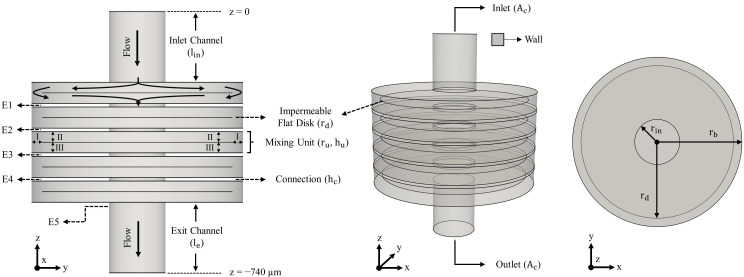
Different views of the 3-D CSFO micromixer geometry. E1, E2, E3, E4, and E5 are the locations 5 µm after the exit of each mixing box.

In this study, a 3-D circular-shaped fluid overlapping (CSFO) passive micromixer is developed. As shown in Figure 1, the micromixer geometry consists of three main branches that are inlet channel, mixing units, and exit channel. The dimensions of the circular inlet and exit channels are equal with a length (*l_i_* and *l_e_*) and cross-section area (*A_c_*) of 200 µm and 2 × 10^4^ µm^2^, respectively. In the CSFO design, five identical mixing units are used to observe the effect of fluid overlapping approach in a wide range of flow conditions. The height (*h_u_*) and radius (*r_u_*) of a single mixing unit are 60 µm and 300 µm, respectively. Each mixing unit is divided equally in the z-direction with a solid, impermeable, and thin-plate disk element which is coaxial with the mixing unit and has a radius (*r_d_*) of 270 µm. It is assumed that the existence of physical joining parts between a disk element and mixing unit will affect the overlapping flow pattern trivially. Therefore, for the sake of designing convenience in the present study, these parts are excluded in the CSFO geometry. In physical applications of the CSFO design, the disk elements can be attached to mixing units from various points as indicated by the line arrows I, II, and III in Figure 1. Other than that, the mixing units are linked to each other via cylindrical extensions, of which height (*h_c_*) is 10 µm and radius (*r_c_*) is equal to that of inlet and outlet channels. Nonetheless, it should be noted that the purpose of including these connection parts is only to be able to measure mixing performance at the outlet of the mixing chambers. In a physical design, these extensions can be omitted since the short residence time of fluids in these sections will contribute to diffusive mixing negligibly. The dimensions of the CSFO micromixer were selected consistent with the 3-D passive micromixer designs studied in the literature. The 3-D CSFO passive micromixer proposed can be fabricated using the current microfabrication technology. Multi-layer fabrication methods [35,36] can be used in physical construction of the CSFO design. Example micromixer studies may be seen in References [21,37,38,39] for detailed fabrication process of 3-D geometries at microscales. 

**Figure 2:**
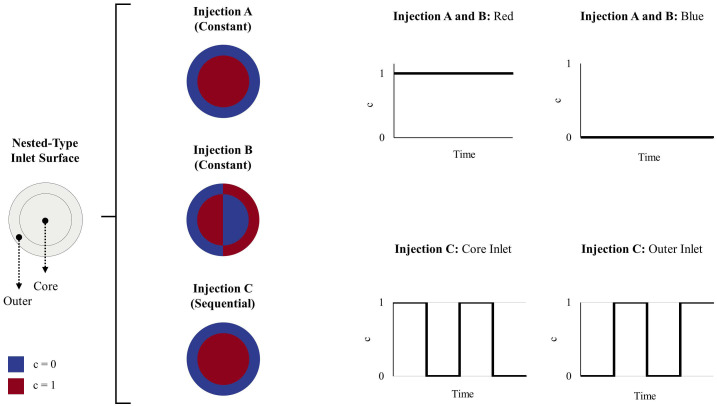
Fluid injection types applied over the inlet boundary of the CSFO micromixer design.

**Figure A1:**
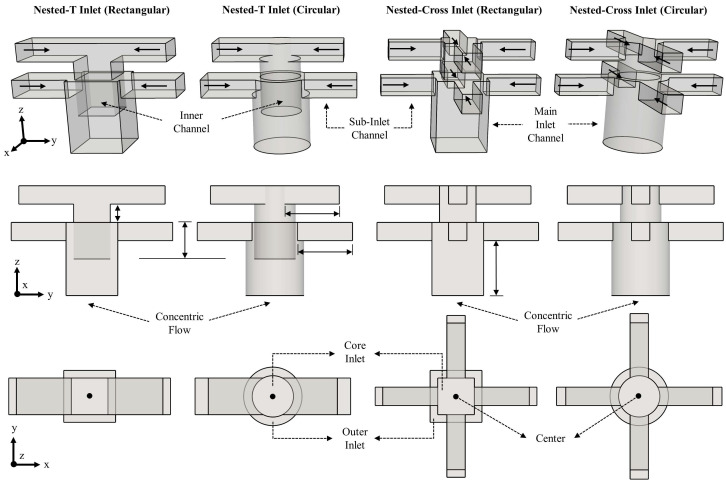
Nested-T (**left two columns**) and nested-cross (**right two columns**) inlet structures to create concentric fluid flow in circular and rectangular microchannels. 3-D view (**top**), yz-plane (**middle**), and xy-plane (**bottom**).

**Figure A2:**
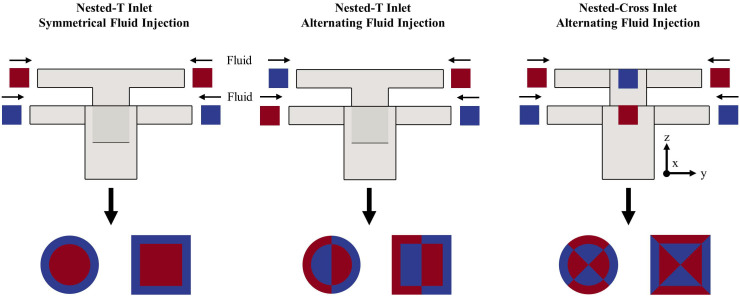
Formation of concentric flows with symmetrical and alternating fluid injections over the nested-t and nested-cross inlet structures. Circular and rectangular planes show the distribution of fluids in concentric flows. Red and blue colors represent different fluids.

In the CSFO micromixer, nested-type inlets are used to create overlapping flow profile throughout the disk surfaces in mixing units. The core and outer segments of inlet surfaces are used to inject fluids as depicted in Figure 2. Please note that these segments have an equal surface area in all injection types applied, and these surfaces are further split equally in injection B. The development of different injection patterns in both circular and rectangular geometries can be seen from Figure A1 and Figure A2 in Appendix A. In the present study, both symmetrical and alternating injection patterns (see Figure 2 and Figure A2) are applied over the inlet boundary. 

**Figure A3:**
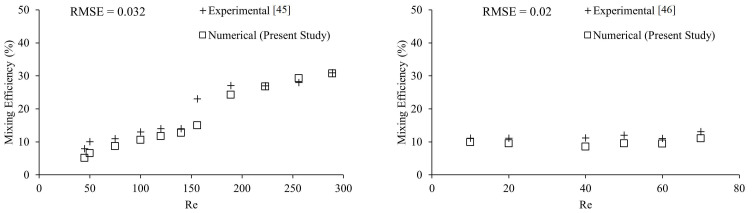
CFD code validation results. Numerical simulation outcomes (present study) against experimental data of two different T-shaped passive micromixers. Root mean square error (RMSE) is used to measure the fit of the numerical simulation results to the experimental data.

Micromixer performance is examined extensively establishing several molecular diffusion constants, i.e., D_1_ = 3.0 × 10^−10^ (crystal violet dye in water [40]), D_2_ = 1.5 × 10^−9^ (fluorescein solution in water [41]) and D_3_ = 3.0 × 10^−9^ m^2^/s (self-diffusion coefficient of water [42]), in a broad range of flow conditions that are Re = 0.1, 0.5, 1, 5, and 10. In all mixing scenarios, equal amount of fluid is injected from each inlet segment in injection A, B, and C. The physical properties of mixing fluids are chosen close to that of water at 20 °C [43,44] with a density (*ρ*) and viscosity (*µ*) of 10^3^ kg/m^3^ and 10^−3^ Pa·s, respectively. To solve the governing flow equations, the following boundary conditions are prescribed in numerical simulations: a uniform velocity profile at the inlets, zero-gauge pressure at the outlet, and zero fluid velocity, i.e., no-slip condition, on solid surfaces. In scalar transport simulations, the gradient of scalar concentration is set to zero at the outlet and solid boundaries to prevent the scalar undergo diffusion over these surfaces. To investigate fluid mixing in the micromixer, relative scalar concentrations, 0 and 1, are imposed on the inlet surface as described schematically in Figure 2. In injection A and B cases, fluid injection is constant over time, and therefore numerical simulations are conducted in two steps as follows. First, a steady-state flow domain is obtained from the simultaneous solution of Equations (1) and (2). Second, a steady-state passive scalar transport simulation is performed by solving the AD equation with the stationary flow domain obtained. In injection C, fluids are injected over the core and outer inlet regions as a square wave with the same injection frequency (f). Thus, a time-dependent simulation is carried out to solve coupled fluid flow and scalar transport equations. In transient simulations, overall simulation times were chosen long enough—for a given flow condition, at least three times of the theoretical fluid mean residence time in the micromixer—to observe the complete development of fluid mixing in the micromixer. In the rest of the paper, CSFO–A, –B and –C notations are used to describe the CSFO micromixer configurations with fluid injection modes A, B and C, respectively. Before the numerical simulations of the test scenarios, the CFD code was validated against the experimental data of two different T-shaped passive micromixer studies in References [45,46]. The validation outcomes, given in Figure A3 in Appendix A, indicate that numerical simulation results are in a good agreement with the experimental data reported in both studies. Thus, the numerical method presented above can be used to predict the mixing performance of the CSFO micromixer proposed. 

## 4. Mesh Study

**Figure A4:**
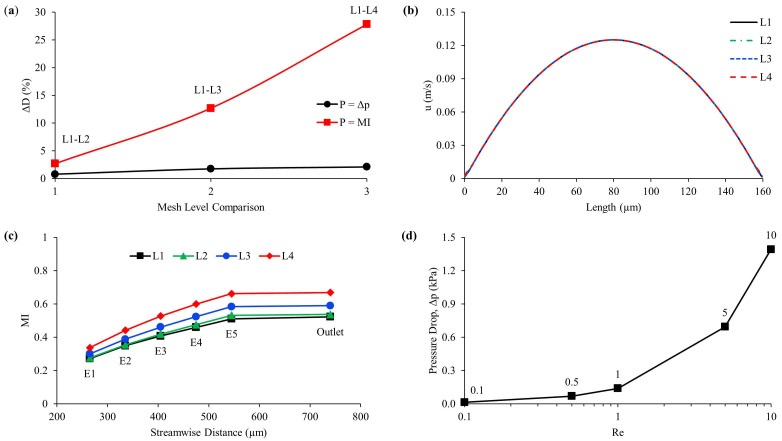
Mesh study outcomes and the change of pressure drop in the CSFO micromixer: (**a**) difference, as a percentage, between L1 and L2, L3, L4 mesh densities with respect to Δp and MI parameters; (**b**) velocity distribution on the diameter of outlet cross-section obtained from L1, L2, L3, and L4 mesh level solutions; (**c**) development of mixing efficiency along the CSFO micromixer in L1, L2, L3, and L4 mesh solutions. MI values are computed on E1, E2, E3, E4, and E5 cross-sections which are normal to the z-direction; (**d**) Δp vs. Re number in the CSFO micromixer.

Numerical solution of advection-dominant transport systems is prone to create high amount of numerical (or false) diffusion errors [34,47]. This is due to inaccurate approximation of steep scalar gradients that inherently develop at high Pe transport conditions. In FVM, maintaining an orthogonality between flow and grid boundaries is pivotal to minimize numerical diffusion. For further information about managing numerical errors in high Pe transport systems, see References [34,47,48,49]. In this research, hexahedron elements are used to discretize the computational domain in numerical simulations. A systematic mesh study is performed by determining four different grid levels in the computational domain of the CSFO micromixer. Total element numbers in L1, L2, L3, and L4 grid levels are 3.90 × 10^6^, 2.45 × 10^6^, 1.58 × 10^6^, and 1.05 × 10^6^, respectively. Numerical simulations are carried out for the highest Pe number scenario examined in the CSFO–B micromixer configuration (i.e., Pe = 3.33 × 10^4^ when Re = 10 and D = D_1_ = 3.0 × 10^−10^ m^2^/s). Mesh study outcomes are presented in Figure A4a–c in Appendix A.

Figure A4a shows that there is a good agreement between the finest and coarser mesh levels when the pressure drop is used to quantify the relative numerical errors in numerical solutions. The maximum variation is calculated as 2.1% between L1 and L4 meshes which evidently indicates that even the coarsest grid level, L4, can provide quite accurate results in fluid flow simulations. The same agreement between different mesh level solutions is also seen in Figure A4b, which shows the distribution of velocity along the diameter of outlet plane. On the contrary, when outlet mixing efficiency is employed in error analysis, numerical solutions exhibit a high divergence as indicated by the rising trendline in Figure A4a. In fact, such a discrepancy between the two trendlines occurs due to quite different transport conditions in fluid flow and scalar transport simulations. While a mild Re number (Re = 10) in the former offers a better control of numerical errors even in relatively coarse grids, the latter is carried out at a very high Pe number (Pe = 3.33 × 10^4^). Hence, much smaller mesh elements are required to approximate sharp scalar gradients accurately. Accordingly, mesh study outcomes need to be evaluated in refence to scalar transport simulations to employ a suitable mesh density in the simulations. For the MI parameter given in Figure A4a, the differences in L1–L4, L1–L3, and L1–L2 comparisons are measured to be nearly 28, 13, and 2.7%, respectively. The lessening percentages indicate that false diffusion generation is suppressed noticeably with increasing mesh densities. The convergent trend of mesh refinement can also be seen in Figure A4c, which shows the development mixing efficiency along the CSFO micromixer for all mesh levels tested. Considering the small variation, i.e., 2.7%, against a large mesh density difference, i.e., 1.45 × 10^6^ elements, between L1–L2 mesh levels, L2 mesh level is determined to conduct numerical simulations. Furthermore, this selection is also validated by estimating an effective diffusivity coefficient from the scalar transport solution of L2 mesh level as proposed in [48]. The ratio of effective diffusivity coefficient to molecular diffusion constant was found to be 1.112 which is quite close to 1. This implies that the molecular diffusion constant simulated is mostly recovered from the numerical solution and the amount of numerical diffusion errors is trivial. Therefore, the use of L2 mesh density provides mostly physical mixing outcomes even in the worst-case scenario. Please note that in other mixing scenarios established in the present paper, numerical solutions will generate much less numerical diffusion due to diminishing magnitude of Pe number in mild transport conditions. 

## 5. Results

### 5.1. Fluid Mixing in the CSFO–A and CSFO–B Micromixer Configurations

**Figure 3:**
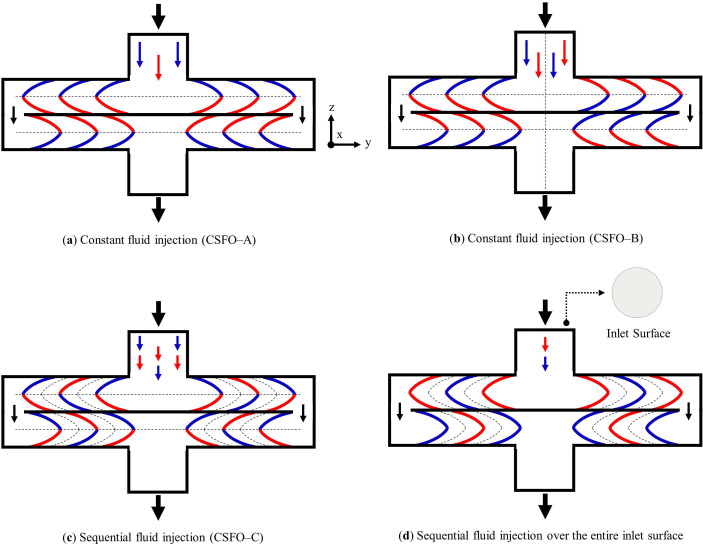
The distribution of injected fluids in each mixing unit of the micromixer configurations (**a**) CSFO–A; (**b**) CSFO–B; and (**c**) CSFO–C. (**d**) The distribution of fluids in a single mixing unit when the entire inlet surface is used to inject fluids sequentially. The dashed lines and curves show the contact surfaces formed between different fluids. Red and blue colors represent the mixing fluids injected from inlet surface(s). Black arrows show flow direction.

**Figure A5:**
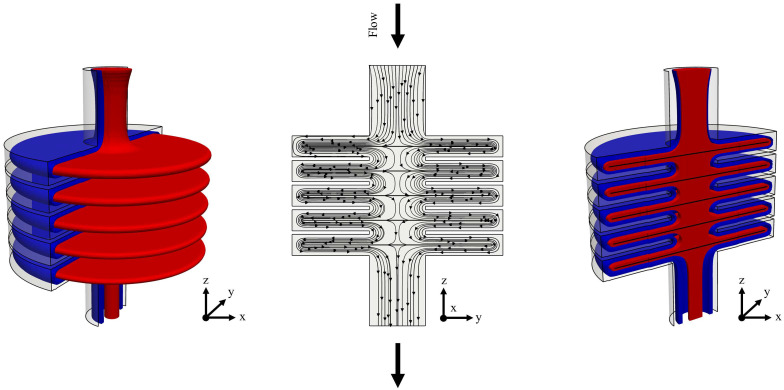
3-D flow domain and flow pathlines in the CSFO micromixer. Fluid overlapping flow profile (**left and right**) and flow pathlines between inlet and outlet on the central plane of the CSFO design (**center**). Red and blue colors show the fluids injected from core and outer inlets, respectively.

At small Re numbers, ineffective manipulation of fluid bodies cause yielding a small contact area between fluid bodies, which in turn limits mixing by diffusion. To overcome this problem and enlarge the interfacial area between mixing fluids, a typical approach is to create several laminations in microchannels [38]. In this method, the main flows are divided into numerous sub-streams or layers of fluid sections which are aligned in microchannels to be in serial or parallel flow regions. In laminating micromixers [37,50,51,52], the overall contact surface is proportional to the number of different fluid segments generated in the micromixer. Although diffusive mixing is promoted over the interfacial area shared by the fluid segments, usually a complex channel network is required to align fluids in microchannels. In the CSFO micromixer design proposed, the enhancement of contact area is ensured without generating multiple flow sectors in the flow domain. Instead, entire fluid bodies are overlapped and stretched in compact mixing units. As can be seen from Figure A5 in Appendix A, which shows the flow pathlines and 3-D flow domain in the CSFO micromixer, fluids that are injected from core and outer inlet segments flow concentrically through the inlet channel and are stretched over the disk surface. During the fluid flow in the CSFO micromixer, the injected fluids occupy different volumes of the flow domain. As the core flow (shown in red in Figure A5) follows a path around disk elements at the central region of the micromixer, the outer flow (shown in blue in Figure A5) develops between the core flow and micromixer walls. Therefore, a quite large interfacial area is yielded between the two fluid bodies due to the encapsulation of the core flow by the outer flow across the CSFO micromixer domain. The development of contact surface in both upper and lower compartments of a single mixing unit is shown schematically in Figure 3. 

As shown in Figure 3 the overlapping (or stratified) fluid pattern expands throughout the disk surface in the upper volume of mixing chamber and flows to the lower volume through the gap between the mixing chamber and the disk element. In the lower section, the above streams are converged at the exit of the cylindrical box and transferred to the next mixing unit. In all design configurations, the same flow cycle is repeated until the fluids are conveyed to the main exit channel of the micromixer. While both CSFO–A and CSFO–B configurations develop a contact surface on the horizontal plane, CSFO–B micromixer also forms an interface in the vertical direction due to alternating fluid injection imposed on the core and outer inlet segments. The horizontal and vertical contact areas formed between mixing fluids are represented by the dashed lines in Figure 3. Meanwhile, it should be noted that the total area of the gap region is approximately 2.7 times higher than that of exit cross-sections. Thus, the fluid flow is not restricted in the gap region and the residence time of fluid particles in a single mixing unit is controlled by the area of the exit cross-section. In the present CSFO micromixer design, the surface area of inlet, outlet, and exit planes are kept equal as noted earlier. 

**Figure 4:**
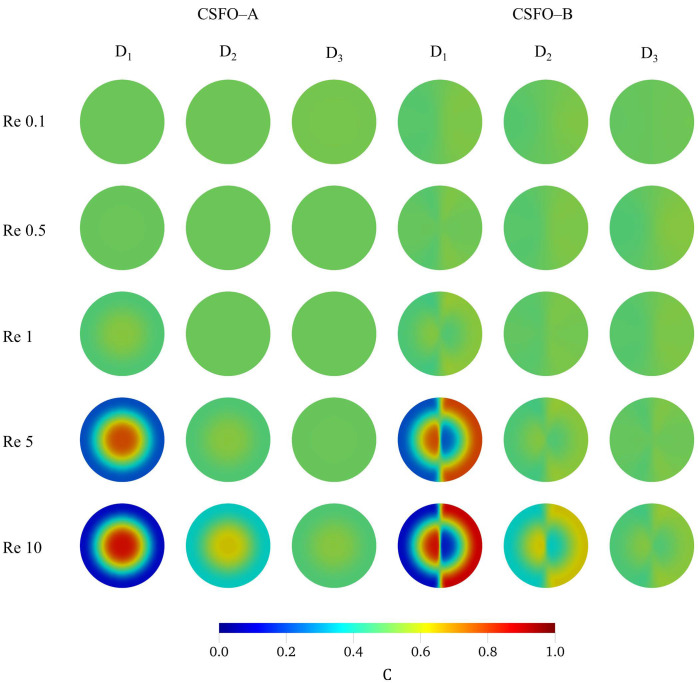
The distribution of scalar concentrations on the exit cross-section of the CSFO–A (first three columns on the left) and CSFO–B (last three columns on the right) micromixer configurations.

**Figure A6:**
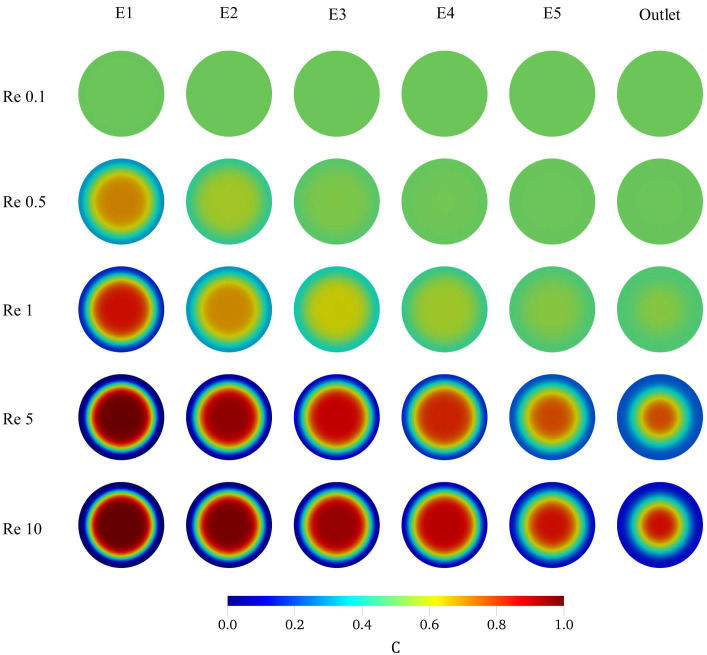
The development of fluid mixing along the CSFO–A micromixer configuration for all flow scenarios of D_1_ diffusion constant. Circular planes show the distribution of scalar concentration on the exit cross-section of mixing units (E1, E2, E3, E4, and E5) and outlet of the CSFO micromixer. E1, E2, E3, E4, E5, and outlet planes are normal to the z-direction.

**Figure A7:**
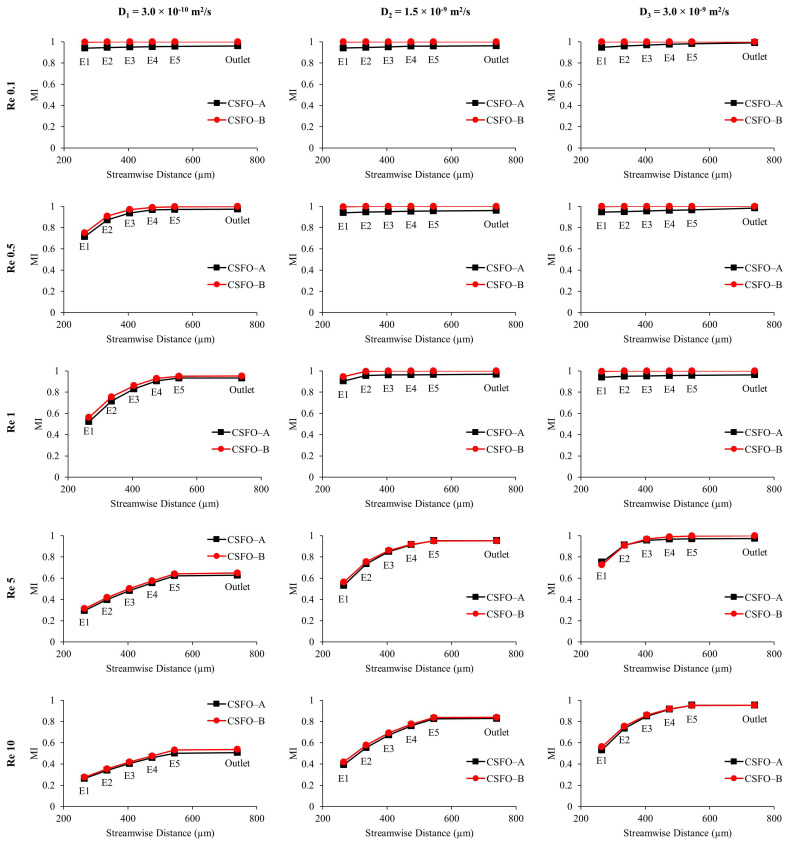
The development of MI along the CSFO–A and CSFO–B micromixer configurations for all flow conditions (i.e., Re = 0.1, 0.5, 1, 5, and 10) and molecular diffusion constants (D_1_, D_2_, and D_3_). MI values are calculated on the E1, E2, E3, E4, and E5 cross-sections which are normal to the z-direction.

When the mixing performance of micromixers are measured, the outcomes evidently show that diffusive mixing—in the vertical direction—is activated across the large interfacial areas formed. Figure A7 in Appendix A shows the development of fluid mixing along the CSFO–A and CSFO–B micromixers for all mixing conditions tested. Firstly, regarding the results in Figure A7, it can be said that the vertical contact surface formed in the CSFO–B micromixer affects the mixing performance trivially. The MI values of both configurations indicates that even the maximum difference is less than 5%. This is because the degree of mixing is mainly controlled by the horizontal surface areas developed in the upper and lower sections of mixing boxes. The contribution of the additional interface to the diffusive mixing—in the horizontal direction—is more visible at low flow conditions, whereas this effect vanishes by lessening residence time of fluid particles at higher Re numbers. In the lowest flow condition (Re = 0.1), almost a complete fluid mixing (MI > 94%) is observed at the exit of the first mixing unit. Moreover, although it is not reflected in the plots, the distribution of scalar concentration in simulation results showed that the biggest portion of the mixing takes place only in the upper section of the first mixing box. In all molecular diffusion scenarios, more than 90% mixing efficiency is yielded in a distance less than 260 µm in the main streamwise direction. At Re = 0.5, while at least two mixing units are required to provide more than 90% mixing value when the smallest diffusion constant is used, this is not the case in higher diffusivity conditions. In D_2_ and D_3_ mixing scenarios, more than 94% mixing efficiency is obtained at the exit of the first mixing unit of the CSFO–A micromixer. As can be pursued from the change of the trendlines in increasing Re numbers, reducing contact time between fluid bodies suppresses the inter–diffusion continually. Hence more mixing units are required to enhance the degree of mixing in the micromixer. Notably, the combination of low contact times with small diffusion coefficients develops the most challenging mixing conditions in the micromixer. At Re = 1, while D_2_ and D_3_ diffusivities can still be tolerated against the fluid residence time reduced, the use of the smallest diffusion constant becomes difficult. In that case, the mixing distance increases to 400 µm (E3) and 470 µm (E4) to obtain nearly 86% and 93% MI, respectively. In all mixing scenarios tested, the lowest mixing efficiencies are obtained for the two highest flow conditions of the D_1_ case as expected. The MI values at the exit of the micromixers are found to be nearly 65% and 54% for Re = 5 and 10, respectively. As mentioned previously, although the contact surface area is increased substantially in CSFO geometry, the development of mixing by diffusion is prohibited by rising flowrates. Nonetheless, the MI values are still promising for the higher molecular diffusion constants, D_2_ and D_3_, as shown in Figure A7. At Re = 5, while D_2_ scenario provides more than 86% mixing efficiency at the exit of the third mixing unit (E3), only two units are required to reach a MI value of nearly 91% (E2) in D_3_ case. For the same diffusivity scenarios, D_2_ and D_3_, the highest flow condition, Re = 10, yields 84% and 92% fluid mixing at E5 and E4 exits, respectively. For all mixing conditions examined, the distribution of scalar concentrations on the outlets of CSFO–A and CSFO–B micromixers are presented in Figure 4. In addition, for the smallest diffusion constant, D_1_, the development fluid mixing on different cross-sections along the CSFO–A configuration can be seen from Figure A6 in Appendix A. 

### 5.2. Fluid Mixing in the CSFO–C Micromixer Configuration

**Figure A8:**
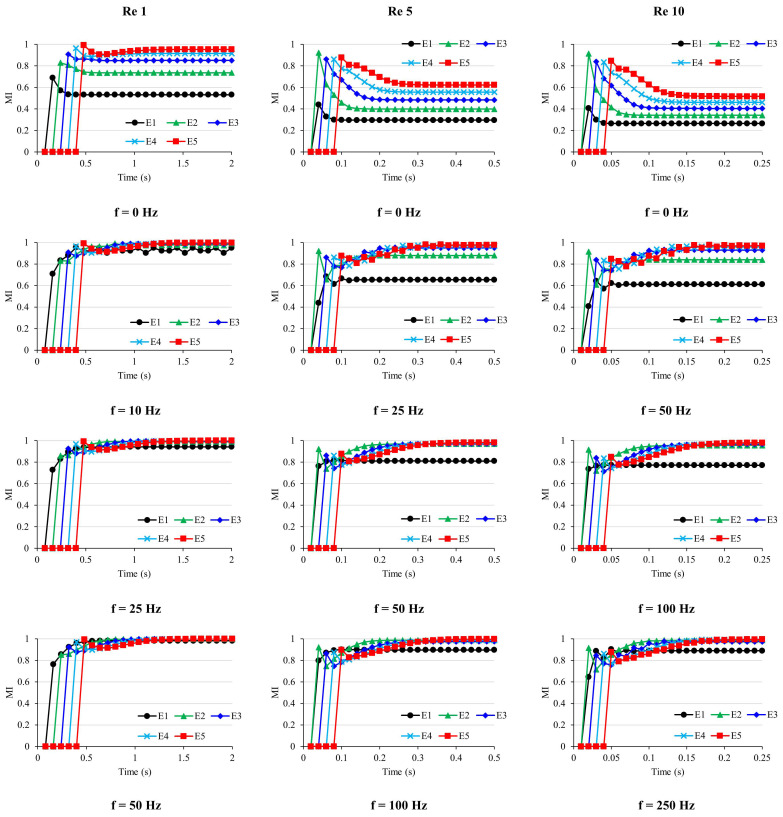
The development of fluid mixing with respect to time at the exit of mixing units in the CSFO–C micromixer configuration when Re = 1, 5, and 10 and D = D_1_. f = 0 Hz plots (**first row**) show time-dependent solutions of CSFO–A micromixer. MI values are calculated on the E1, E2, E3, E4, and E5 cross-sections which are normal to the z-direction.

As discussed above, when the CSFO micromixer is operated with constant fluid injection, large contact surfaces are yielded between fluid bodies in the horizontal directions. However, in the CSFO design, the overall interfacial area can be enhanced further if the fluids are injected sequentially over the core and outer inlet segments as described in Figure 2. Sequential or pulse injection of fluids can be achieved by manipulating micropumps as described and used in References [53,54,55,56]. In the case of sequential injection in CSFO–C configuration, the development of additional contact areas between consecutive fluid pairs is enabled as shown schematically in Figure 3c (see the dashed curves). In mixing units, these new interfaces move dynamically by expanding and shrinking in the upper and lower mixing sections, respectively, which creates a wave pattern throughout the disk surfaces. Therefore, in each half volume of the mixing units, diffusive mixing is also promoted in the horizontal directions. It should be noted that unlike the CSFO–A and CSFO–B configurations, where entire fluid bodies are overlapped on the horizontal plane, in the CSFO–C micromixer, different fluid segments develop the overlapped fluid structure due to wave pattern in the horizontal direction. The mixing performance of the CSFO–C configuration is investigated in various injection frequencies—between 10 and 250 Hertz (Hz) depending on the flow condition—for the most challenging mixing scenarios (i.e., D = D_1_ and Re = 1, 5, and 10). The evolution of mixing efficiencies is observed with respect to time at the exit of each mixing unit. The results are plotted in Figure A8 in Appendix A. Please note that in Figure A8, f = 0 Hz plots show time-dependent numerical solutions of CSFO–A micromixer, in which fluid injection is constant over time as described in Section 3 and Figure 2. These solutions are used to compare the relative effects of constant and sequential fluid injections in the CSFO design.

Figure A8 evidently shows that the formation of additional contact surface areas accelerated diffusive mixing substantially. At Re = 1, even the lowest injection frequency, f = 10 Hz, is adequate to reduce the mixing distance (MI > 90%) to the exit of the second mixing unit (E2). However, further increase of the injection frequency contributes to the overall mixing efficiency slightly. When the mixing outcomes are compared with that of CSFO–A micromixer, CSFO–C configuration (f = 10 Hz) provides a rapid fluid mixing over a very short distance. To reach a MI value around 85%, the time and distance required are “240 millisecond (ms) and 330 µm” and “640 ms and 400 µm” in CSFO–C and CSFO–A configurations, respectively. Therefore, the use of sequential injection reduces mixing time and distance by the factors of 2.7 and 1.2, respectively. Much higher improvements in mixing values are seen in Re = 5 and 10 flow conditions as indicated by the rising trendlines in Figure A8. Meanwhile, it needs to be explained that before reaching their steady values, the mixing efficiencies follow a declining and rising trend after a sharp increase at early stages. The spikes in the trendlines are observed at the exit of each mixing unit in both CSFO–A and CSFO–C micromixer configurations. These peak points essentially occur due to the following reason explained. At the beginning of the fluid flow in the inlet channel, the formation parabolic flow profile yields a relatively high contact area and diffusive mixing starts developing on this surface. During the fluid flow, the diffusive mixing on the parabolic front travels in the micromixer and leaves the micromixer in the end. Therefore, the peak mixing efficiency, which is generated at the very early stage, is observed at the exit of the mixing units. After the peak values of MI, the declining and rising trends show the actual development of mixing efficiency in the micromixers.

At Re = 5, as constant fluid injection (f = 0 Hz) can only offer a MI value around 63% (t = 280 ms) at E5 location, more than 85% MI (t = 120 ms) is obtained at the exit of the second mixing unit (E2) by the use of sequential fluid injection in the CSFO geometry (f = 25 Hz). For higher injection frequencies, f = 50 and 100 Hz, the degree of mixing rises to 95% (t = 160 ms) and 98% (t = 180 ms) levels at the same location (E2), respectively. Unlike the Re = 1 flow condition, the effect of injection frequency is more visible at Re = 5. While f = 25 Hz case provides nearly 65% (t = 140 ms) mixing efficiency at the exit of the first mixing box (E1), the MI values reach 81% (t = 120 ms) and 90% (t = 100 ms) levels in f = 50 and 100 Hz scenarios, respectively. In the highest flow scenario, Re = 10, while nearly 53% MI (t = 140 ms) can be measured at the last exit location (E5) of the CSFO–A micromixer, CSFO–C configuration provides more than 61% MI (t = 70 ms) at the exit of the first mixing unit (E1) for the lowest injection frequency tested (f = 50 Hz). When the injection frequency is set to f = 100 and 250 Hz, the MI value reaches 77% (t = 80 ms) and 88% (t = 100 ms) on the same exit location (E1), respectively. As the best-case scenarios at Re = 5 (f = 25 Hz) and Re = 10 (f = 50 Hz), CSFO–C configuration develops approximately 85% (t = 120 ms) and 83% (t = 70 ms) mixing efficiencies in a distance less than 330 and 400 µm, respectively. When these mixing figures are compared with the outputs of the CSFO–A configuration, mixing conditions are improved significantly in terms of efficiency, distance, and time. Notably, such an improvement could be achieved by means of the extra contact areas formed between consecutive fluid segments during the sequential injection.

## 6. Discussion

The CSFO micromixer and nested-type inlets developed in this study offer a novel design approach to mix fluids at microscales. Unlike the conventional micromixer designs, where the enhancement of interfacial area strongly depends on the effective manipulation of fluid flow in microchannels, the CSFO geometry inherently develops a large contact area without requiring a complex flow formation in the micromixer domain. Therefore, better operating conditions are obtained. As can be seen from Figure A4d, the CSFO design improves fluid mixing under reasonable pressure drop conditions. Even the highest flow condition, Re = 10, yields a pressure drop value of less than 1.4 kPa, which is quite acceptable compared to that of reported in the literature [57,58]. The pressure values in Figure A4d can be decreased further when the number of mixing units are reduced in the design. When the mixing performance of the CSFO micromixer is compared with other studies in the literature, a substantial amount of mixing efficiency is achieved over a very short distance as presented in Table 1. 

It should also be noted that the use of nested-type inlets is not only limited to the CSFO micromixer, but also can be used in any type of active or passive micromixer designs. Concentric flows that are developed in nested-type inlets basically provide two main advantages. First, when the fluids are injected concentrically, the deformation of fluid bodies in the micromixer becomes relatively much easy compared to the conventional fluid injections in separate channels. For instance, in split-and-recombination (SAR) micromixers [43,61,62], several mixing units are required to increase the distribution of inlet streams in sub-channels. When, however, fluids are injected concentrically, the distribution ratio of different fluids in the sub-channels is increased, and hence the number of mixing units required can be reduced. Second, the nested-type inlets inherently create a contact area between the two fluids being injected. Thus, fluid mixing is initiated at the beginning of the inlet channel before fluids reach to the micromixer. The test simulations, which we do not report here, showed that the use of concentric flows in circular or rectangular channels improves diffusive mixing significantly when Re ≤ 0.1. Therefore, in extremely slow flow conditions, only a straight or curved channel with a nested-type inlet can be used as a micromixer.

The CSFO micromixer can also function without employing the nested-type inlets when the fluid injection is sequential. In such a case, the entire inlet surface is used to feed the micromixer with different fluids sequentially. However, in this condition, the interfacial area on the horizontal plane is not formed and the overall contact surface is developed by the wave pattern as described schematically in Figure A4d. Besides this function, the CSFO geometry can be modified to be operated at much higher flow conditions by generating chaotic advection in the micromixer. For this purpose, the disk elements can be redesigned with alternative grooves or obstacles to create complex flow patterns in the mixing units. 

**Figure 5:**
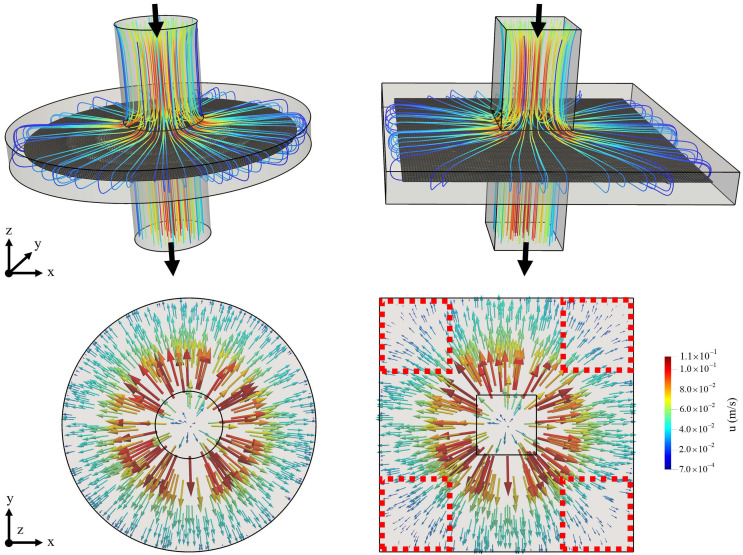
The distribution of flow vectors and flow pathlines in single–mixing–box circular (**left**) and square (**right**) fluid overlapping design configurations (Re = 10). Black arrows show flow direction.

In addition to the circular micromixer design, the fluid overlapping mixing approach can also applied in rectangular or polygonal (e.g., pentagon, hexagon, etc.) geometries. However, when a rectangular geometry is used, a non-uniform velocity distribution can develop on the rectangular plane that divides mixing box volume equally. As displayed in Figure 5 which shows the flow pathlines and the distribution of flow vectors in single-mixing-box circular and square designs (Re = 10), the two geometries render varying flow profiles. In contrast to smooth flow distribution in the circular design, the fluid flow is dominated at the center of the horizontal directions in the square geometry, which creates dead flow zones at the corner regions of the square box (see the dashed red lines). Although that variation in the flow structure does not affect the development of the fluid overlapping pattern in the mixing box, the diffusive interaction is diminished. That is due to the yield of a relatively a smaller contact area and increased flow velocity in central directions, which reduces contact time. Regarding the outcomes in Figure 5, the circular geometry appears to be an optimal shape for the fluid overlapping mixing approach.

Consequently, considering the plain design structure and high mixing performance, the CSFO passive micromixer can be integrated with microfluidic systems or used as a stand-alone device to mix fluids at microscales. 

## 7. Conclusions

In this research, fluid overlapping mixing approach and nested-type inlets are introduced for passive micromixers. A 3-D circular-shaped passive micromixer design is developed to enhance fluid mixing particularly at low flow conditions that is Re < 10. The mixing performance of the CSFO micromixer is examined numerically in various fluid flow and molecular diffusion conditions. The effects of alternative design configurations and injection strategies are tested. Numerical simulation results indicate that the CSFO design creates a large contact surface between mixing fluids in both upper and lower volumes of each mixing unit. In the case of constant fluid injection, the overlapping fluid pattern develops an interfacial area throughout the disk elements on the horizontal plane. However, when the fluids are injected sequentially, additional contact areas are formed between consecutive fluids. While symmetrical and alternating fluid feeding types provide almost identical results in the constant injection scenarios, the mixing effect of injection frequency is increased with rising Re numbers in the sequential injection cases. In both injection conditions, high mixing efficiency values could be achieved with a reasonable pressure drop in the CSFO micromixer. The maximum pressure drop is found to be less than 1.4 kPa at Re = 10. For the smallest diffusion coefficient and constant fluid injection, more than 90% mixing efficiency is quantified in a distance of 260, 400, and 470 µm for Re = 0.1, 0.5, and 1 flow scenarios, respectively. The mixing distances are reduced further even in high flow conditions when fluids are injected sequentially. When the mixing outcomes are compared with that of reported in the literature, the CSFO design offers a high amount of fluid mixing over a very short distance. Therefore, the CSFO micromixer can be employed in next generation microfluidic systems, where short mixing distances will be required, to mix fluids at microscales.

## Figures and Tables

**Table 1:** The comparison of the CSFO–A configuration with the micromixers reported in the literature in terms of mixing performance in low flow conditions (Re < 10).

Micromixer	Re	Mixing Efficiency (%)	Mixing Length (µm)	Reference
Crossing Channels	0.1	88	6400	[57]
Multi-Inlet	0.1–0.29	90–80	5000	[26]
Serpentine	0.2	100	7500	[59]
Baffled	0.29	52	7200	[25]
T-Shaped (f = 20 Hz)	0.3	86.5	500	[27]
T-Shaped (split inlet)	0.5	42	2000	[18]
Vortex	0.5	50	1000	[21]
Rhombic	1	55	6000	[58]
Obstructed Channels	1	55	1180	[60]
CSFO–A (D_1_)	0.1	94	260	Present study
	0.5	94	400	
	1	91	470	

## Data Availability

Data is contained within the article.
